# Cardiomyocyte microvesicles: proinflammatory mediators after myocardial ischemia?

**DOI:** 10.1007/s11239-020-02156-x

**Published:** 2020-06-14

**Authors:** Patrick Malcolm Siegel, Judith Schmich, Georg Barinov, István Bojti, Christopher Vedecnik, Novita Riani Simanjuntak, Christoph Bode, Martin Moser, Karlheinz Peter, Philipp Diehl

**Affiliations:** 1 Cardiology and Angiology I, Heart Center Freiburg University, Medical Faculty, University of Freiburg, 79106 Freiburg im Breisgau, Germany; 2 Baker Heart & Diabetes Institute, Atherothrombosis & Vascular Biology Laboratory, Melbourne, Australia; 3 Faculty for Medicine & Nursing, Monash University, Melbourne, Australia

**Keywords:** C-reactive protein, Myocardial infarction, Microvesicles, Cardiomyocytes

## Abstract

Myocardial infarction is a frequent complication of cardiovascular disease leading to high morbidity and mortality worldwide. Elevated C-reactive protein (CRP) levels after myocardial infarction are associated with heart failure and poor prognosis. Cardiomyocyte microvesicles (CMV) are released during hypoxic conditions and can act as mediators of intercellular communication. MicroRNA (miRNA) are short non-coding RNA which can alter cellular mRNA-translation. Microvesicles (MV) have been shown to contain distinct patterns of miRNA from their parent cells which can affect protein expression in target cells. We hypothesized that miRNA containing CMV mediate hepatic CRP expression after cardiomyocyte hypoxia. H9c2-cells were cultured and murine cardiomyocytes were isolated from whole murine hearts. H9c2- and murine cardiomyocytes were exposed to hypoxic conditions using a hypoxia chamber. Microvesicles were isolated by differential centrifugation and analysed by flow cytometry. Next-generation-sequencing was performed to determine the miRNA-expression profile in H9c2 CMV compared to their parent cells. Microvesicles were incubated with a co-culture model of the liver consisting of THP-1 macrophages and HepG2 cells. IL-6 and CRP expression in the co-culture was assessed by qPCR and ELISA. CMV contain a distinct pattern of miRNA compared to their parent cells including many inflammation-related miRNA. CMV induced IL-6 expression in THP-1 macrophages alone and CRP expression in the hepatic co-culture model. MV from hypoxic cardiomyocytes can mediate CRP expression in a hepatic co-culture model. Further studies will have to show whether these effects are reproducible in-vivo.

## Highlights

- Microvesicle release from cardiomyocytes is increased during hypoxia
- Cardiomyocyte microvesicles contain a distinct set of miRNA from their parent cells
- CMV contain inflammation associated miRNA
- CMV stimulate IL-6 expression in THP-1 macrophages
- CMV stimulate CRP expression in a hepatic co-culture model

## Introduction

Cardiovascular disease and myocardial infarction in particular, are the leading cause of mortality worldwide [1]. Inflammation plays a major role in the immune response after myocardial infarction [2]. Elevated serum-CRP-levels after myocardial infarction are associated with “harmful inflammation” and poor outcomes [3, 4]. As a part of the acute phase response [5], CRP is produced by hepatocytes following stimulation by IL-6 from Kupffer cell macrophages lining the liver sinusoids [6, 7]. Elevated IL-6 levels have been reported after myocardial infarction [8]. However, the exact mechanisms leading to an increase in IL-6 and CRP after myocardial infarction have not been sufficiently characterised.

Microvesicles (MV) are small membrane vesicles released by most cell types upon activation and apoptosis. They are loaded with bioactive molecules including proteins, mRNA, microRNA which are distinct from their parent cells and can alter the function of their target cells rendering them *intercellular mediators* [9–11].

*Cardiomyocyte* microvesicles (CMV) are released under stress during myocardial infarction or hypoxia [12, 13]. They have been shown to contain proinflammatory cytokines such as TNF-α [14], DNA, mRNA [15] and various miRNA [16] which they protect from degradation. Multiple functions have been attributed to CMV, for example delivery of distinct miRNA [13] to fibroblasts or monocytes after myocardial infarction and protection of the heart from ischemia reperfusion injury [17].

**Fig. 1:**
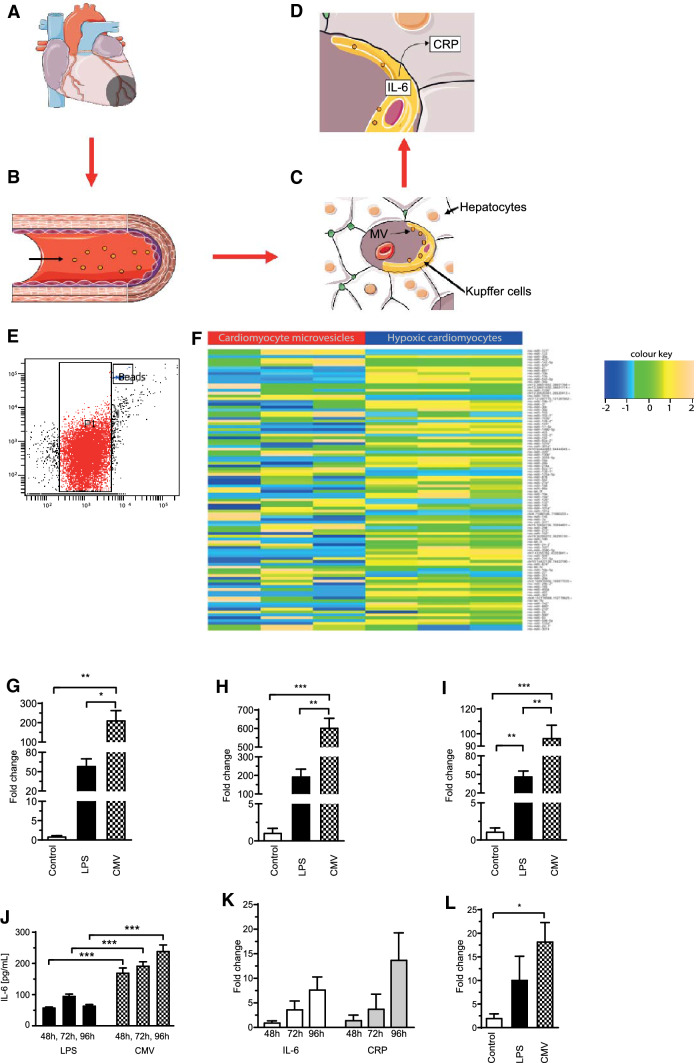
Suggested model of CMV-mediated CRP expression in hepatocytes. For more information see text, underlying images of Fig. 1a–d were adapted from http://smart.servier.com/ under a creative commons licence (**a–d**). Microvesicles were successfully isolated from the supernatant of hypoxic H9c2 cardiomyocytes. P1 indicates the microvesicle gate, “beads” indicates a gate for standardized counting beads (**e**). Next-generation sequencing revealed that CMV contain a distinct pattern of miRNA compared to their parent cells as shown by the heat map. The color key indicates the z-score of the normalised read-count of individual miRNAs. Most miRNAs had higher read counts in hypoxic cells than in CMV (**f**). IL-6 expression was upregulated as shown by qPCR using RNA from THP-1 macrophages after incubation with H9c2 CMV for 48 (**g**), 72 (**h**) or 96 h (**i**). Induction of IL-6 expression by CMV in THP-1 macrophages was verified on a protein level by ELISA after 48, 72 and 96 h. IL-6 levels were increased after incubation with CMV even compared to the positive control (LPS) (**j**). CMV induced a gradual rise in IL-6 and CRP expression in the co-culture of HepG2 and THP-1 cells as determined by qPCR (**k**). CRP increase was also higher compared to the negative control exemplified after 96 h (**l**)

In the following, we would like to suggest a *theory* of how CMV may lead to increased hepatic CRP production after myocardial infarction. It is intended to explain to the reader why we performed the in-vitro studies described in the results section: After myocardial infarction (Fig. 1a) hypoxic cardiomyocytes release CMV into the circulation (Fig. 1b) which are loaded with a distinct set of pro-inflammatory mediators including miRNA. After reaching the hepatic circulation CMV are first taken up by Kupffer cells (specialized macrophages in the liver) lining the walls of the sinusoids (Fig. 1c). There, they promote IL-6 expression which, in a paracrine manner, then induces CRP expression in hepatocytes (Fig. 1d).

The aim of this study was to investigate the effects of CMV from hypoxic cultured cardiomyocytes on hepatic CRP expression.

## Materials and methods

### Cell culture and stimulation

THP-1 cells, HUVEC and H9c2 (immortalized rat myoblasts [18]) (ATCC, USA) and HepG2 (immortalized hepatocytes, CellBank, Australia) were cultured using standard protocols.

Before stimulation, cells were seeded in 6-well plates and incubated for 72 h. THP-1 cells were differentiated into macrophages using PMA (200 ng/mL). Cells were stimulated with isolated CMV or PBS for different durations followed by RNA-extraction and qRT-PCR. THP-1 cells were also stimulated with LPS (15 µg/mL) as a positive control.

### Co-culture of HepG2 and THP-1 cells and stimulation

We created a simplified model of the liver by co-culturing THP-1 macrophages and HepG2 liver cells. THP-1 cells were differentiated to macrophages and washed before HepG2 cells were added. After incubation for 72 h, co-cultures were stimulated with CMV, LPS (15 µg/mL), PBS or medium followed by RNA-extraction and qRT-PCR.

### Isolation of murine cardiomyocytes

Isolation of murine cardiomyocytes was performed as described elsewhere [19]. Briefly, male C57BL/6 mice were euthanized, the heart was surgically removed and connected to a Langendorff perfusion system followed by perfusion with a collagenase solution for up to 50 min. The heart was transferred into petri-dishes, the atria were removed, and undigested parts of the heart muscle were eliminated using a filter. Isolated cardiomyocytes were then cultured in laminin-coated (Sigma-Aldrich, USA) dishes using minimal essential medium (MEM, Gibco^®^, USA) supplemented with 2.5% FBS and 50U/mL penicillin and 50 µg/mL streptomycin.

### Measurement of troponin-T

Troponin-T was quantified using the Cobas 8000 Modular Analyser (Roche, Switzerland).

### Isolation of CMV after cardiomyocyte hypoxia

7 × 10^5^ H9c2-cells or 1 × 10^5^ murine cardiomyocytes were placed in the hypoxia chamber (Billups-Rothenberg Inc., USA) flooded for 10 min with a nitrogen-enriched mixture of gases containing 1.5% O_2_ and 5% CO_2_ and incubated within this hypoxic atmosphere for 6 h. Controls were cultured under normoxic conditions.

CMV were isolated by differential centrifugation. Approximately 8 × 10^5^ CMV from H9c2 or 2 × 10^5^ CMV from murine cardiomyocytes were added to cells for stimulation experiments.

### Flow cytometry

CMV from H9c2 -cells or murine cardiomyocytes were diluted in PBS and counted by flow cytometry. An MV gate was defined using Megamix-Plus Beads (BioCytex, France) to include MV with a size range between 0.2 and 1 µm. 10,000 counting beads (Trucount, BD, USA) were recorded per sample and the MV concentration was calculated.

Vitality of cardiomyocytes was assessed using propidium iodide staining (PI, R&D Systems, USA) as described previously [20].

### RNA extraction and qPCR

RNA was isolated and transcribed to cDNA according to standard protocols. ∆∆C_t_ values were computed and fold change (FC) was determined as follows: fold change = 2−^∆∆Ct^.

### Next-generation sequencing

RNA-samples from CMV and hypoxic H9c2 were subjected to next-generation sequencing (NGS) to detect every known as well as possible new miRNAs within these samples and NGS was performed as described previously [11].

### Statistics

Differences between multiple means were assessed by one-way ANOVA. When only two columns are depicted in graphs a paired t-test was used to assess for difference of means, since treated and control samples were always handled in parallel. p < 0.05 was considered statistically significant (Asterisks: *p < 0.05, **p < 0.01, ***p < 0.001). All experiments were performed at least threefold. Analysis was performed using GraphPad Prism 8.31 and figures were prepared for submission using Adobe^®^ Illustrator^®^ CS6. Statistical analysis of next-generation sequencing experiments was performed as described previously [11].

## Results

### Hypoxic H9c2-cells release MV and contain a distinct set of pro-inflammatory miRNA

After incubation in the hypoxia chamber, CMV from H9c2-cells were successfully isolated from the cell supernatant as shown by flow cytometry (Fig. 1e).

Next-generation sequencing revealed that the miRNA-profile from H9c2-cells and their respective CMV differed significantly (Fig. 1f). The abundance of miRNA in cells compared to CMV was quite different as most miRNA had a higher abundance in cells than in CMV.

In the hypoxic *cell* samples 38 miRNA had more than 10,000 read counts and 6 more than 100,000, while in *CMV* the highest read-count was 7,661 (miR-143) and all in all there were only 9 miRNA with a read-count greater than 1,000. The five most abundant miRNA in *CMV* were miR10a_2, miR10b_4, miR22_1, miR143_1 and miR423_5p). Interestingly the miRNA profile of hypoxic and non-hypoxic H9c2-*cells* only differed in one miRNA: miRNA-210, which is known to be upregulated during hypoxia [21]. However, miRNA-210 only showed low read counts in CMV (< 1000).

Several miRNA involved in inflammation or immunity were also detected in CMV and are displayed in Table 1.

**Table 1:** Overview of the miRNAs known to be involved in inflammation and immunity upregulated in H9c2 CMV

Name	Read count CMP	Full name
miR-21	916	mmu-miR-21-5p
miR-26	294	mmu-miR-26a-5p
miR-30	65	mmu-miR-30a-5p
miR-126	78	mmu-miR-126-5p
miR-132	11	mmu-miR-132-3p
miR-143	7661	mmu-miR-143-3p
miR-146	53	mmu-miR-146a-5p
miR-181	1866	mmu-miR-181a-5p

### CMV from hypoxic H9c2-cells induce IL-6 expression in THP-1 macrophages and CRP expression in a hepatic co-culture model

Incubation of CMV from H9c2 with THP-1 macrophages led to an increase in IL-6 expression (FC IL-6 after 48 h, 72 h, 96 h: LPS: 59.59 ± 10.27, 196.7 ± 37.05, 47.68 ± 7.90 vs. CMV: 214.1 ± 48.47, 605.9 ± 49.16, 96.86 ± 10.03, p < 0.05; Fig. 1g–i). Increased IL-6 expression by THP-1 macrophages after CMV stimulation was verified on a protein level by an IL-6 ELISA (IL-6 [pg/mL] after 48 h, 72 h, 96 h: LPS: 59.38 ± 1.12, 96.85 ± 5.04, 65 ± 2.72 vs. CMV: 171.3 ± 14.29, 193.6 ± 11.74, 240.7 ± 18.78, p < 0.001, Fig. 1j). Moreover, CMV effected a gradual increase of IL-6 and CRP expression in *co-cultures* over time (FC after 48 h, 72 h, 96 h: IL-6 1.07 ± 0.28, 3.76 ± 1.62, 7.80 ± 2.48; CRP 1.57 ± 0.94, 3.88 ± 2.89, 13.85 ± 5.42, Fig. 1k). Furthermore, CRP expression in *co-cultures* was increased compared to the negative control (cell culture medium) after 96 h (FC CRP after 96 h: negative control 2.10 ± 0.82, CMV 18.32 ± 3.94, p < 0.05, Fig. 1l).

### Hypoxia leads to CMV release from murine cardiomyocytes

**Fig. 2:**
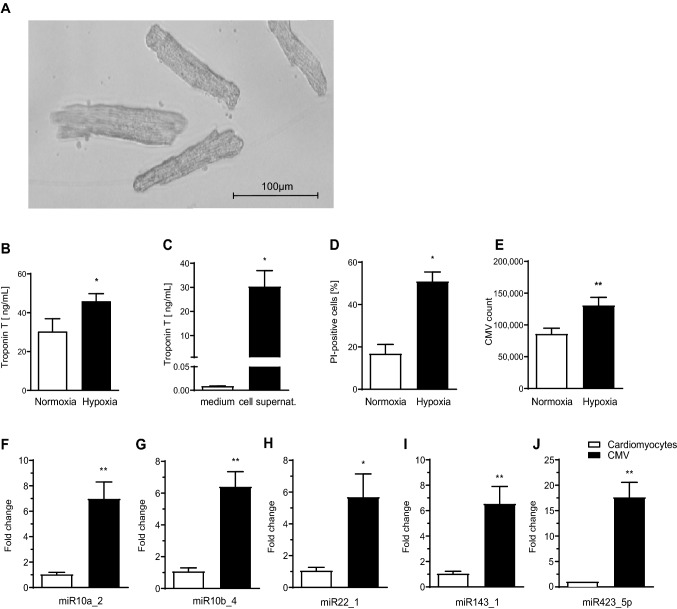
Murine cardiomyocytes were successfully isolated and cultivated from whole murine hearts visualized by light transmission microscopy (**a**). Increased levels of troponin-T were recorded in the supernatant of cardiomyocytes exposed to hypoxia (**b**). Contamination of cell culture medium with troponin-T was ruled out (**c**). Exposure to hypoxia led to significantly increased cardiomyocyte death compared to normoxic conditions as shown by PI staining in flow cytometry (**d**). More CMV were released from murine cardiomyocytes under hypoxic conditions and counted by flow cytometry (**e**). Murine CMV displayed a similar miRNA profile as H9c2 CMV. qPCR showed that the 5 most abundant miRNA in H9c2 CMV were also upregulated in murine CMV (black columns) compared to cardiomyocytes (white columns) (**f–j**)

We aimed to verify the results gained from the eternal cell line H9c2 using primary murine cardiomyocytes. Isolation of murine cardiomyocytes was successful (Fig. 2a) and approx. 2.5 × 10^5^ cardiomyocytes were isolated from a murine heart. Exposure of murine cardiomyocytes to hypoxia led to increased levels of troponin-T in the cell supernatant (troponin-T [ng/mL]: hypoxia 45.95 ± 3.88 vs. normoxia 30.30 ± 6.65, p < 0.05, Fig. 2b). Contamination of the cell culture medium by troponin-T in FBS was ruled out (troponin-T [ng/mL]: medium 0.01 ± 0.00 vs. cell supernatant of normoxic cardiomyocytes 30.30 ± 6.65, p < 0.05, Fig. 2c). Hypoxia increased the percentage of dead cardiomyocytes as identified by propidium iodide PI staining (PI positive cells [%]: normoxia 16.80 ± 4.40 vs. hypoxia 50.87 ± 4.50, p < 0.05, Fig. 2d). Furthermore, hypoxic cardiomyocytes released more CMV compared to their normoxic counterparts as determined by flow cytometry (CMV count x 10^3^: normoxia 85.75 ± 9.21 vs. hypoxia 130.61 ± 12.85, p < 0.01, Fig. 2e).

### The miRNA profile of CMV from *murine* cardiomyocytes is comparable to CMV from H9C2-cells

The most abundant miRNA in CMV from H9c2-cells were also upregulated in murine CMV compared to their parent cells indicating a similar miRNA profile (FC expression CMV vs. hypoxic cardiomyocytes: miR10a_2: 7.00 ± 1.31 vs. 1.05 ± 0.14, miR10b_4: 6.42 ± 0.93 vs. 1.09 ± 0.20, miR22_1: 5.70 ± 1.44 vs.1.07 ± 0.20, miR143_1: 6.55 ± 1.35 vs. 1.06 ± 0.17, miR423_5p: 17.64 ± 2.90 vs. 1.06 ± 0.17, p < 0.01—p < 0.05, Fig. 2f–j).

### CMV from hypoxic *murine* cardiomyocytes also lead to increased CRP expression in a hepatic co-culture model

**Fig. 3:**
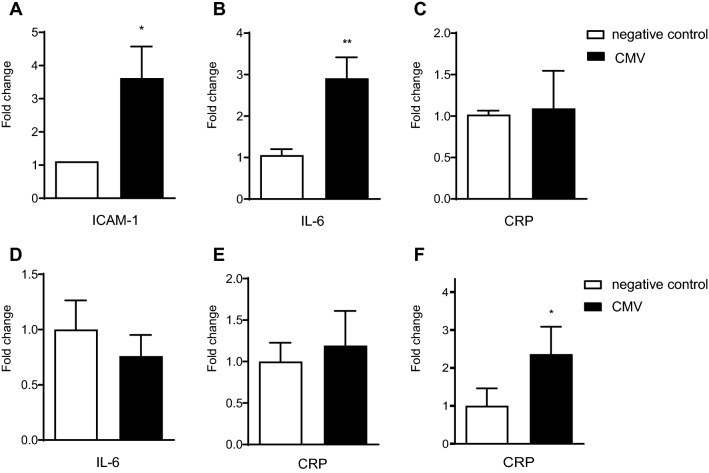
Biological capacity of murine CMV was first assessed on HUVEC. Increased ICAM-1 expression was shown by qPCR after incubation of CMV with HUVEC (**a**). Murine CMV induced upregulation of IL-6 in a monoculture of THP-1 macrophages (**b**) but did not affect CRP expression in these cells alone (**c**) as shown by qPCR. Incubation of CMV with a monoculture of HepG2 cells did not affect either IL-6 or CRP expression determined by qPCR (**d, e**). However, in a co-culture of HepG2 hepatocytes and THP-1 macrophages CRP expression was elevated by CMV as determined by qPCR (**f**)

Initial experiments to assess biological capacity of CMV demonstrated that incubation of CMV with HUVEC for 18 h led to activation evidenced by increased ICAM-1 expression (FC expression of ICAM-1, CMV vs. negative control (PBS): 3.62 ± 0.95 vs. 1.12 ± 0.26, p < 0.05, Fig. 3a). Incubation of CMV with THP-1 macrophages for 24 h led to an upregulation of IL-6. CRP-expression, however, was not affected by CMV after incubation with THP-1 macrophages alone (FC expression CMV vs. negative control (PBS): IL- 6: 2.91 ± 0.51 vs. 1.06 ± 0.14, p < 0.01, CRP: 1.01 ± 0.46 vs 1.02 ± 0.05, p = 0.87; Fig. 3b, c). Moreover, CMV incubation with HepG2 cells alone for up to 72 h did not affect IL-6 or CRP expression (FC expression CMV vs. negative control (PBS): IL-6: 0.76 ± 0.20 vs. 1.00 ± 0.26, p = 0.16, CRP: 1.19 ± 0.42 vs. 1.00 ± 0.23, p = 0.71, Fig. 3d, e). However, the incubation of CMV with a co-culture of HepG2 and THP- 1 cells for 72 h resulted in an increase of CRP expression compared to the negative control (FC CRP expression CMV vs. negative control (PBS): 2.40 ± 0.72 vs. 1.00 ± 0.45, p < 0.05, Fig. 3f).

## Discussion

In this study we aimed to prove our hypothesis that CMV released from hypoxic cardiomyocytes induce hepatic CRP expression. We were able to isolate CMV from both H9c2- and murine cardiomyocytes after incubation in the hypoxia chamber (Figs. 1e and 2a, e). The hypoxia chamber is intended to simulate the conditions cardiomyocytes are exposed to during the ischemia after myocardial infarction and it has successfully been applied by other researchers to generate CMV [22, 23]. Increased cardiomyocyte death and rising levels of troponin-T are evidence that hypoxia was achieved (Fig. 2b–d).

MV can transfer miRNA between their host and target cells [24]. We therefore chose to explore the miRNA content of CMV using next-generation sequencing. It provides strong advantages over microarrays including higher sensitivity and a wider miRNA spectrum that can be analysed [25]. It revealed a changed miRNA profile in H9c2 CMV compared to hypoxic H9c2-cells (Fig. 1f) which suggests a selective packaging mechanism previously described for stimulated THP-1 cells and their MV [11].

CMV from hypoxic H9c2 cardiomyocytes were rich in inflammation-affecting miRNA (Table 1). The most abundant miRNA in this group, miR-143, is increased in ischemic areas of the heart after myocardial infarction and has been shown to promote cardiac fibrosis by targeting the “Sprouty-3” gene in cardiomyocytes [26]. Moreover, increased levels of miR-143 in monocytes after myocardial infarction correlate with increased hsCRP-levels [27]. It could be hypothesized that increased uptake of CMV containing miR-143 by monocytes induces IL-6 expression which later leads to increased CRP expression by hepatocytes.

miR-181 has been reported to alter the myocardial response to oxidative stress by targeting “mt-COX-1” or “PTEN” [28]. miR-21 is also upregulated in the infarcted zone after myocardial infarction and promotes cardiac fibrosis by inhibition of the “Smad7” pathway [29]. The role of miR-21 in inflammation and its relation with CRP is emphasized by the fact that both elevated circulating miR-21 and increased CRP-levels after myocardial infarction are predictive of left ventricular remodelling [30]. Additionally, miR-146, which was also upregulated in CMV has been shown to increase IL-6 expression by promoting the “NFκB” pathway in an in-vitro sepsis model [31].

Little is known on the miRNA profile of CMV of mouse compared to rat cardiomyocytes. Since next-generation sequencing was only performed for H9c2 CMV we showed that the most abundant miRNAs of H9c2 CMVs were also upregulated in mouse CMV (Fig. 2f–j). As with H9c2 CMV, we therefore assume a distinct miRNA pattern of *mouse* CMV from their parent cells, *murine* cardiomyocytes.

To investigate the effects of CMV on the acute phase response in the liver, we successfully established a co-culture of THP-1 macrophages and HepG2 hepatocytes. This strategy is superior to monoculture of HepG2 cells since it creates a more physiologic model of the liver [32, 33]. Both, CMV from hypoxic H9c2-cells and murine CMV stimulated co-cultures to express CRP (Figs. 1l and 3f). Induction of an acute phase response in the liver by MV has also been reported by other groups, for example, by murine brain-derived microvesicles after brain injury [34].

HepG2 cells alone did not react to stimulation with CMV (Fig. 3e) with an increase in CRP production. We therefore hypothesized that there might be a paracrine mechanism via Kupffer cells. To elucidate the effects of CMV on macrophages, THP-1 macrophages alone were incubated with H9c2- or murine CMV and stimulation of IL-6 expression was found (Figs. 1g–j and 3b). Other groups have shown that microvesicles can induce pro-inflammatory effects on target cells [11, 35, 36]. We claim this supports our model of IL-6 production by hepatic Kupffer cells after stimulation by CMV.

Although increased levels of IL-6 have been reported after myocardial infarction [37] the major source remains unclear. Shu et al. claimed IL-6 after myocardial infarction was also produced in the vulnerable plaque and necrotic myocardium [38]. We cannot rule this out but argue it does not contradict our model since CMV may also induce IL-6 synthesis in a paracrine manner in myocardial macrophages.

Peak circulating levels of CRP after myocardial infarction correlate with infarct size, development of heart failure, and mortality [4, 39]. This may be due to pro-inflammatory effects of CRP itself. More recently it has been found that microvesicles from patients after myocardial infarction can convert *pentameric* CRP into pro-inflammatory *monomeric* CRP [40, 41]. In light of these findings our study gains importance as it provides an explanation for the rise in CRP-levels after myocardial infarction. Moreover, it offers an opportunity to target “harmful” inflammation *upstream* at the beginning of the inflammatory cascade. Therapeutic options could include inhibiting CMV release after myocardial infarction using low doses of the Ca^2+^-Channel inhibitor Verapamil [42], inhibiting MV uptake by target cells using Annexin V [43] or by inhibiting specific miRNAs using specialized antagomirs [44].

Additionally, miRNA containing CMV may serve as novel biomarkers for the detection of myocardial infarction since they protect miRNA from degradation [45]. miRNAs which were detected in CMV in our study and have been reported as biomarkers in the literature include miR-21, miR-30 and miR-133 [46].

We would like to acknowledge the limitations of our study. The effect of cardiomyocyte MV on hepatic CRP production was not investigated in-vivo and should be confirmed in a mouse model. The exact mechanisms of how miRNA lead to increased CRP expression was not assessed and could be determined using antagomir experiments.

In summary, we demonstrated that CMV released from cardiomyocytes during hypoxia contain a distinct miRNA profile which differs from their parent cells. Furthermore, CMV were able to induce CRP expression in a liver co-culture model of HepG2 and THP-1 cells.

## Data Availability

All data is available from the authors upon reasonable request. Not applicable.
